# Feasibility and Safety of Outpatient Thyroidectomy: A Narrative Scoping Review

**DOI:** 10.3389/fendo.2021.717427

**Published:** 2021-07-28

**Authors:** Justine Philteos, Elif Baran, Christopher W. Noel, Jesse D. Pasternak, Kevin M. Higgins, Jeremy L. Freeman, Albino Chiodo, Antoine Eskander

**Affiliations:** ^1^Department of Otolaryngology–Head & Neck Surgery, University of Toronto, Toronto, ON, Canada; ^2^Undergraduate Department of Psychology, University of Toronto, Toronto, ON, Canada; ^3^Institute of Health Policy, Management and Evaluation, Dalla Lana School of Public Health, University of Toronto, Toronto, ON, Canada; ^4^Division of General Surgery, Department of Surgery, University of Toronto, Toronto, ON, Canada

**Keywords:** thyroidectomy, endocrine surgery, ambulatory surgery, de-escalation, same-day discharge, outpatient procedures

## Abstract

**Background:**

Outpatient thyroid surgery is gaining popularity as it can reduce length of hospital stay, decrease costs of care, and increase patient satisfaction. There remains a significant variation in the use of this practice including a perceived knowledge gap with regards to the safety of outpatient thyroidectomies and how to go about implementing standardized institutional protocols to ensure safe same-day discharge. This review summarizes the information available on the subject based on existing published studies and guidelines.

**Methods:**

This is a scoping review of the literature focused on the safety, efficacy and patient satisfaction associated with outpatient thyroidectomies. The review also summarizes and editorializes the most recent American Thyroid Association guidelines.

**Results:**

In total, 11 studies were included in the analysis: 6 studies were retrospective analyses, 3 were retrospective reviews of prospective data, and 2 were prospective studies. The relative contraindications to outpatient thyroidectomy have been highlighted, including: complex medical conditions, anticipated difficult surgical dissection, patients on anticoagulation, lack of home support, and patient anxiety toward an outpatient procedure. Utilizing these identified features, an outpatient protocol has been proposed.

**Conclusion:**

The salient features regarding patient safety and selection criteria and how to develop a protocol implementing ambulatory thyroidectomies have been identified and reviewed. In conclusion, outpatient thyroidectomy is safe, associated with high patient satisfaction and decreased health costs when rigorous institutional protocols are established and implemented. Successful outpatient thyroidectomies require standardized preoperative selection, clear discharge criteria and instructions, and interprofessional collaboration between the surgeon, anesthetist and same-day nursing staff.

## Introduction

### Background

The American Thyroid Association (ATA) has recently released an interdisciplinary consensus statement with regards to outpatient thyroidectomies. They investigated the peri-operative factors that optimize safe performance of ambulatory thyroidectomies (1). Traditionally, thyroidectomies were an inpatient procedure for close monitoring of potential post-operative complications, however, increasingly, outpatient thyroidectomy has been adopted albeit with large variations in practice (2). The primary objective of this article is to provide a scoping review and rationale for the feasibility and safety of outpatient thyroidectomies, the patient-selection factors required for safe outpatient thyroidectomy, and the implementation of hospital programs that make outpatient thyroidectomies practicable.

### Thyroidectomy Complications

Thyroidectomy is a relatively low risk procedure; however, the potential post-operative complications include: symptomatic hypocalcemia due to parathyroid gland injury, airway compromise secondary to cervical hematoma, and recurrent laryngeal nerve injury (3, 4). The incidence of these complications is quite low (5–10).

Intraoperative injury to the parathyroid glands can result in post-operative hypoparathyroidism and, consequently, symptomatic hypocalcemia. A single 1-hour post-thyroidectomy parathyroid hormone level has been validated to risk stratify patients into low-risk and high-risk groups for developing post-operative symptomatic hypocalcemia (11–16). Early calcium and vitamin D supplementation for patients in the high-risk category can effectively prevent symptomatic hypocalcemia (17). Outpatient bloodwork and close follow up is preferred by patients over inpatient management (18). In the age of integrated electronic patient care, surgeons can follow serum calcium values upon discharge and adjust calcium supplementation accordingly. As such, hypocalcemia is rarely a reason to avoid outpatient thyroidectomy given (1) our ability to predict it and (2) our ability to manage it with oral supplementation and with close ambulatory follow up.

There is also the risk for paresis or paralysis of the recurrent laryngeal nerve. Hospital admission for suspected unilateral recurrent nerve injury is not required as the majority of patients tolerate recurrent laryngeal nerve injury well. These injuries are often transient. In approximately 50% patients there is full return of function within the first 6 months (19). Furthermore, if concern for recurrent laryngeal nerve injury arises intra-operatively, intra-operative nerve monitoring and a post-operative nasopharyngoscopy examination can confirm the movement of the vocal cords. Expectant management and patient education with regards to swallowing precautions can be provided accordingly if vocal cord paralysis is noted (9, 18). Bilateral vocal cord paresis is extremely rare, often predicted based on intra-operative findings and nerve monitoring.

Lastly, the most feared complication of thyroid surgery is the risk of a post-operative hematoma resulting in airway compromise; however, recent literature quotes post-thyroidectomy hematoma rates of 0.25%-1.0%. Furthermore, 40-50% of hematomas are reported to occur within 6 hours post-operatively, and less than 10% occur beyond the first 24 hours (20–23). Thus, with mandated set length-of-time for observation, rapidly-progressive arterial hematomas are unlikely to occur after a patient is discharged home. After propensity score matching was used to match patients who were discharged on postoperative day 0 and those discharged on postoperative days 1 or 2, a retrospective study analyzing 10, 502 patients who underwent thyroidectomies found that there was no difference in hematoma rates requiring intervention for patients who were discharged same-day versus those who were admitted overnight - for patients who suffered a hematoma requiring further intervention, such as drainage, operation, or tracheostomy, in both crude rates (0.8% *vs* 0.5%, P = .16) and adjusted odds (OR 1.81, 95% CI 0.81–4.31) for those discharged POD0 and POD1-2, respectively (24). Retrospectively-evaluated patient-factors associated with post-operative hematoma have contributed to a predictive scoring system intended to identify patients at higher risk for this complication (3, 19–21, 25–27). With the implementation of predictive risk-stratification, and established discharge protocols, these patients are flagged for hematoma evaluation prior to discharge, which mitigates the risk of airway obstruction. Ultimately, the surgeon must judge the adequacy of hemostasis at the end of the operation to decide whether same-day discharge is appropriate.

### Same Day Surgery

The rationale for pursuing outpatient thyroidectomy is founded on the fact that thyroidectomies are often well-tolerated by patients (28). Potential post-operative complications can be risk-stratified prior to same-day discharge, and appropriate intervention and follow-up can be implemented accordingly. The aforementioned post-operative complications can be identified and observed prior to discharge. With regards to the risk of post-operative hematomas, more than half will occur within the initial 6-hour post-operative period – which is still within the window of same-day surgery observation as most centers observe patients for 8-12 hours prior to discharge (29, 30). This is further exemplified in the literature that demonstrates no increased incidence of complications that might have been prevented by a prolonged postoperative inpatient stay (31). High volume centers have reported good outcomes with even shorter hospital stays of less than 3 hours’ postoperative observation (8). Thus, in experienced hands, outpatient thyroidectomies are safe. Clear discharge instructions and reasons to return to the emergency department mitigate the risk of later occurring complications.

### Patient Satisfaction and Cost Effectiveness

Outpatient thyroidectomies are valued by patients who prefer post-operative recovery from the comfort of their homes. Studies investigating patient satisfaction with same-day thyroidectomies boast response rates over 80% with high patient satisfaction (26). Patient preference and security in outpatient thyroidectomies were also measured by Sahmkow et al. (32) and reported average scores of 9.3/10 (10 being highest satisfaction and security). They thus concluded that the majority of patients felt safe to leave the hospital after their operation. Clear communication between healthcare providers and their patients prior to discharge was correlated with positive questionnaire responses and no reported displeasure for early discharge (10, 32–36). Given these findings, it is evident that patients have a preference towards post-operative recovery at their own comfort and convenience with clear instructions on reasons to return to the hospital to ensure their safety (10, 36–39).

Outpatient thyroidectomies have been gaining desirability on an institutional level as they are cost-effective and demonstrate conservation of scarce resources (2). In a cost analysis study conducted by Terris et al, it was found that healthcare cost for outpatients was significantly lower than inpatients on average – highlighting a cost reduction of approximately $2,500 USD per patient (10, 40, 41). Thus, in the appropriately-selected patient, outpatient thyroidectomies have been shown to not only optimize perioperative patient experiences but have also demonstrated cost savings.

## Methods

A scoping review study design was employed to examine the full breadth of knowledge with regards to the safety of outpatient thyroidectomies across study designs; as well as, identify gaps in knowledge to inform future research directions and hospital protocol implementation.

A scoping review – aimed to map the research surrounding the literature on outpatient thyroidectomies - was conducted in line with the framework of Arksey and O’Malley (42). The following databases were searched from inception through the Ovid search interface: Medline (1946 – April 20, 2021), Embase (1947 – April 20, 2021), and PsycINFO (1809 – April 20, 202). The searching process followed the Cochrane Handbook (43) and the Cochrane Methodological Expectations of Cochrane Intervention Reviews (MECIR) (44) for conducting the search, the PRISMA guideline for reporting the search (45), and the PRESS guideline for peer reviewing the search strategies (46).

Inclusion criteria comprised randomized controlled trials (RCTs), and observational studies. All English language full-text articles conducted on humans were included regardless of the age of participants. The study outcomes of interest pertained to the criteria employed to determine eligibility for outpatient thyroidectomy.

Data extraction was performed using a unique form created specifically for the purposes of this review. For each included study, study design, eligibility criteria for outpatient thyroidectomy and contraindications for outpatient thyroidectomy were extracted.

## Results

### Search Results

In total, 11 studies were identified and used for this scoping narrative review. In terms of study design, 6 studies were retrospective analyses, 3 were retrospective reviews of prospective data, and 2 were prospective studies.

We aimed to analyze the both the patient factors and tumor characteristics that these studies used to determine their eligibility criteria for a patient’s fitness for outpatient thyroidectomy. The results of which can be found in **Table 1**.

**Table 1:** An overview of the literature analyzing patient characteristics amenable to outpatient thyroidectomy.

AUTHOR (YEAR)	INCLUSION CRITERIA	EXCLUSION CRITERIA	STUDY DESIGN – SAMPLE SIZE
COUNTRY			
**Chin et al. (2007) (**5**)** China	1. Hemithyroidectomies for thyroid nodule size 4 cm and below	1. Large retrosternal goiters	Retrospective n= 114
	2. Patient’s fitness for surgery	2. Goitres causing tracheal obstruction or deviation	
	3. Postoperative family support.	3. Patients who were class three and above on the scale of the American Society of Anesthesiologists’ Physical Class System (ASA)	
		4. Patients who could not meet the general criteria for day surgery procedures.	
**Snyder et al. (2010) (**8**)** USA	1. Cooperative patient interested in outpatient surgery		Prospective n= 1,241
	2. Absence of significant medical comorbidities		
	3. No anticoagulant treatment or need for drain		
	4. No concomitant procedures (e.g. lateral neck dissection)		
	5. Sufficient patient autonomy and social support		
**Terris et al. (2007) (**9**)** USA		1. Significant comorbid conditions	Retrospective review of prospective data n= 91
		2. Patients who underwent concomitant procedures requiring admission	
		3. Patients who expressed a preference for admission	
		4. Patients who required drains - large lesions incurring a potential for significant postoperative dead space	
**Bergenfelz et al. (2008) (**19**)** Sweden	1. Patient within a 1-hour drive from the hospital for at least 48 hours		Retrospective n= 3,660
	2. Adequate support at home		
	3. Hemi-thyroidectomies and subtotal thyroidectomies completed by 13:00 to allow adequate time to monitor		
**Almeida et al. (2010) (**47**)** Portugal		1. Rejection of ambulatory regimen by the patient	Retrospective review of prospective data n= 100
		2. Lack of motivation for an outpatient procedure	
		3. Cognitive disability or low educational level that could not permit an early recognition of the alert signs of a major complication	
		4. Home distance from hospital over 20 km,	
		5. Lack of adequate home facilities	
**Mazeh et al. (2012) (**48**)** USA		1. Comorbidities that may require longer observation owing to the nature of their underlying disease	Retrospective review of prospective data n= 608
		2. Patients who live >3 hours away	
		3. Patients with no individuals to stay with at home	
**Lo Gerfo et al. (1991) (**49**)** USA	1. Patients emotionally and intellectually capable of understanding the procedure and post-operative plan		Retrospective n= 134
**Teoh et al. (2008) (**50**)** China	1. Age <70 years		Retrospective n= 50
	2. ASA grade I/II		
	3. BMI <30 kg/m2		
	4. Benign unilobular thyroid disease		
	5. Willingness to undergo surgery on a day case basis		
	6. Responsible adult available to care for the patient postoperatively in the first 24 hours		
	7. Easy access to a telephone		
**Champault et al. (2009) (**51**)** France		1. ASA of III or IV	Retrospective n= 95
		2. Anticoagulation treatment	
		3. The presence of sleep apnea	
		4. Age >75 years	
		5. Patient lives >50 km from the hospital	
		6. No functional telephone line	
		7. The absence of an adult willing to accompany them home and to stay with them overnight.	
**Sklar et al. (2011) (**52**)** Canada	1. A partial thyroidectomy was performed		Retrospective n= 247
	2. Patients must live or be staying within 20 minutes of the hospital		
	3. Must have a responsible adult at home the first night after the surgery		
	4. ASA risk score of III or less with a		
	stable medical condition		
**Sahmkow et al. (2012) (**32**)** Canada	1. Hemithyroidectomy, total thyroidectomy and completion thyroidectomy	1. History of coagulation problems or current aspirin use	Prospective n= 200
	2. Thyroid surgery +/- central neck dissection	2. Severe comorbidity or uncontrolled systemic disease	
		3. simultaneous surgery	
		4. Lack of social support	
		5. Housing >1 hour drive from hospital	

### Required Patient Characteristics for Outpatient Thyroidectomy

There were three main themes that emerged from the literature with regards to patient characteristics that make them suitable for outpatient thyroidectomy: fitness for surgery, hospital accessibility and social support. With regards to fitness for surgery, two of the included studies used the American Society of Anesthesiologists’ (ASA) Physical Class System to determine a cut-off. The ASA score is determined by pre-anesthetic medical co-morbidities. An ASA score of II or below indicates a patient with mild systemic disease (53). One study indicated a criterion of ASA risk score of III or less with a stable medical condition (52), while the other indicated an ASA score of I/II (50). The other three studies that indicated fitness for surgery as a criterion for outpatient thyroidectomy did not set standardized specifications; but rather, cited “absence of significant medical comorbidities” and “patient’s fitness for surgery” as criteria (5, 8, 32). The second consideration that emerged was patient accessibility. Three studies outlined defined a time cut-off for hospital accessibility (19, 32, 52), and a third required that patients have access to a telephone (50). Lastly, six studies made mention of adequate home support as a requirement for outpatient thyroidectomy (5, 8, 19, 32, 50, 52).

### Patient Characteristics Contraindicated for Outpatient Thyroidectomy

Patient comorbidities were the greatest prohibitor for an outpatient thyroidectomy. Two studies noted an ASA score of III or above as a contraindication for outpatient surgery (5, 51). Other studies reported broader criteria with regards to a patient’s comorbidities precluding them from outpatient thyroidectomy (47, 48). Lastly, social support (48, 51) and distance from hospital (47, 48, 51) were again noted as important factors in stratifying patients’ eligibility for outpatient thyroidectomy. Similarly, a recent retrospective review of thyroidectomies through the American College of Surgeons National Surgical Quality Improvement Program found that patients with dyspnea (at rest or with exertion), a bleeding disorder (including active anticoagulation), or undergoing dialysis were to be excluded from outpatient procedures (54).

### Tumor Characteristics Contraindicated to Outpatient Thyroidectomy

Only one study incorporated tumor characteristics in their exclusion criteria for outpatient thyroidectomy. Chin et al. excluded patients with (a) large retrosternal goiters and (b) goiters causing tracheal obstruction or deviation, from eligibility for outpatient thyroidectomy (5). Furthermore, Terris et al. excluded patients who underwent larger resections as they incurred a potential for significant postoperative dead space which required drain insertion and an admission (9).

### Tumor Characteristics Amenable to Outpatient Thyroidectomy

Six of the included studies identified the final pathologic diagnosis of patients undergoing outpatient thyroidectomy (7–10, 48, 52). Furthermore, a recent meta-analysis concluded that malignant pathology was identified in 322 outpatient thyroidectomy cases (23%) (55). In contrast, one of the included study’s criterion required benign unilobular thyroid disease for outpatient thyroidectomy consideration (50); another study only included hemithyroidectomies with a set cut-off of thyroid nodule size 4 cm and below, regardless of pathology, for consideration of outpatient thyroidectomy (5). With regards to concomitant procedures, Snyder et al. and Sahmkow et al. included patients undergoing procedures such as central neck dissections and parathyroidectomies for consideration of outpatient thyroidectomy. Their studies also included patients undergoing hemithyroidectomies, subtotal thyroidectomies, total thyroidectomies and completion thyroidectomies (8, 32). Lastly, a recent retrospective review also concluded that lesser estimated intraoperative blood loss was also amenable to same-day discharge as it likely signified a less extensive operation (56).

## Discussion

**Figure 1:**
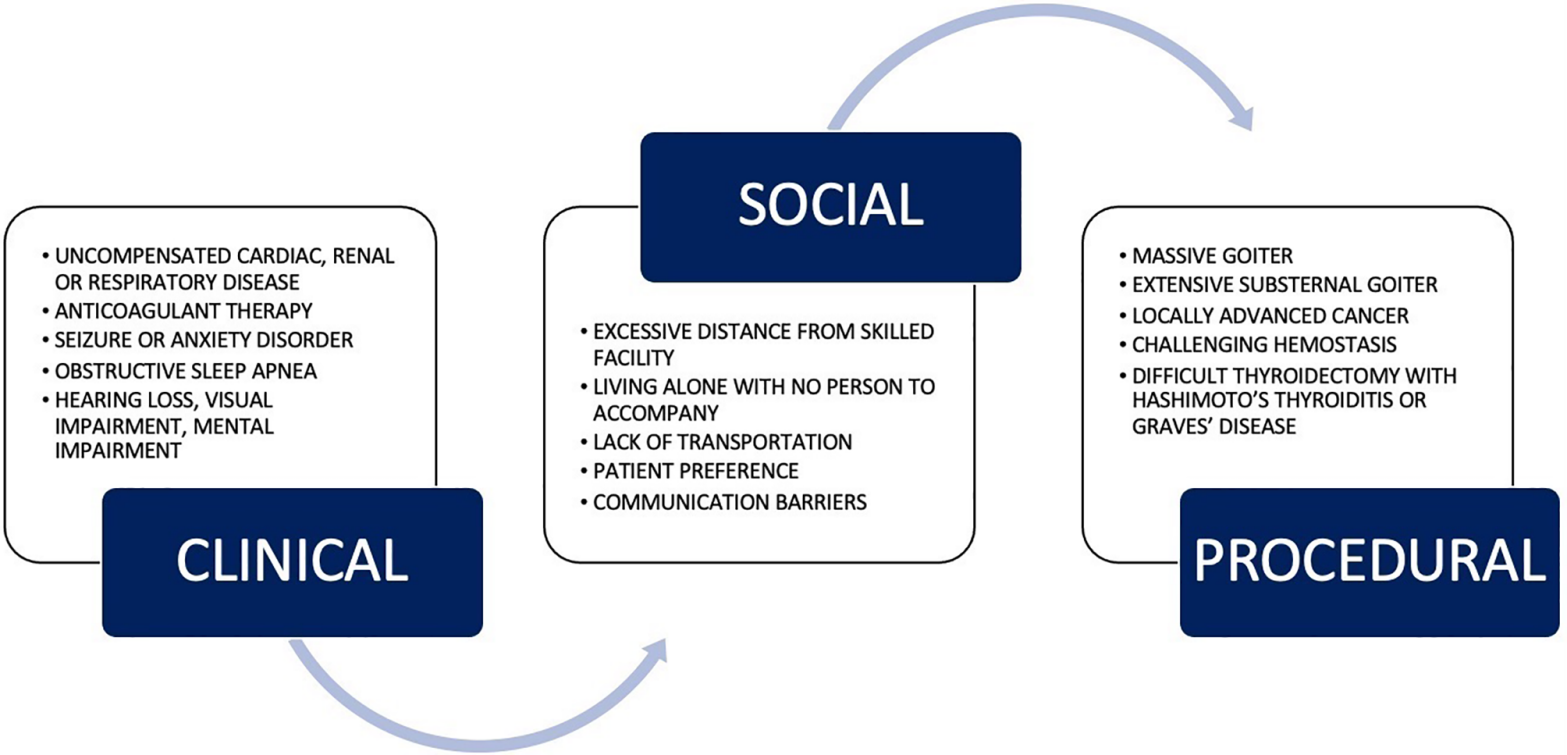
Relative contraindications to outpatient thyroidectomy as outlined by American Thyroid Association. Adapted from the ATA guidelines (1).

**Figure 2:**
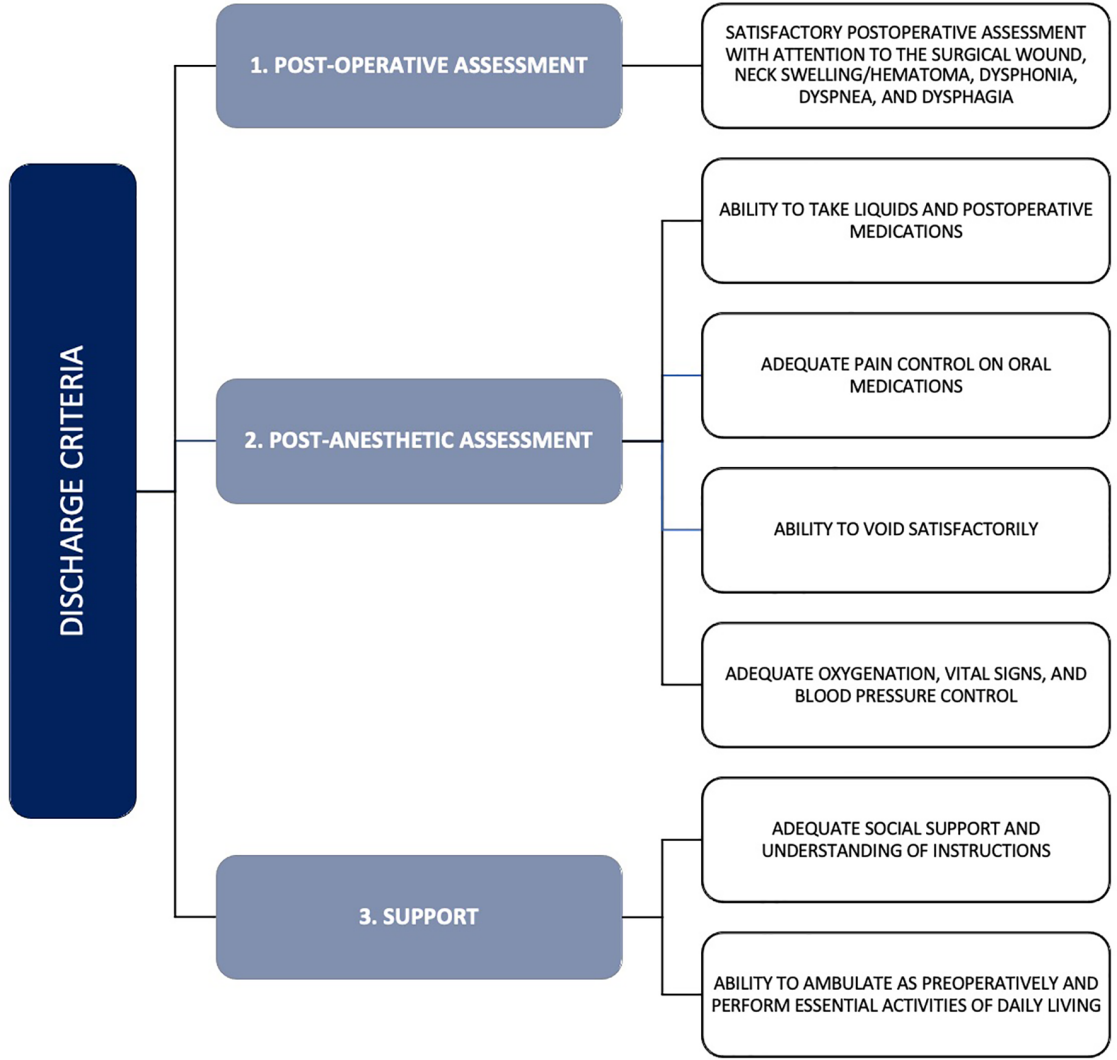
Discharge criteria for outpatient thyroidectomy as outlined by the American Thyroid Association. Adapted from the ATA guidelines (1).

Post-operative discharge planning after thyroidectomy is not one size fits all. It depends on experienced clinical judgment that takes into account the underlying health of the patient, extensiveness and difficulty of the anticipated thyroidectomy, risk for postoperative bleeding, and other patient characteristics (8). The ATA released their statement on outpatient thyroidectomies – its indications, contraindications, and discharge criteria – in 2013 (1). **Figure 1** outlines the contraindications to outpatient thyroidectomies as defined by the ATA (1); furthermore, their criteria set to facilitate safe discharge after same-day thyroidectomy is outlined in **Figure 2**. The recommendations from the ATA and the papers reviewed above can be summarized into three broad categories that need to be satisfied to facilitate safe outpatient thyroidectomy: pre-operative patient comorbidity, social support, and extent of thyroid disease and its resection (1).

Successful outpatient thyroidectomies require rigorous pre-operative selection, discharge criteria and instructions, and interprofessional collaboration between the surgeon, anesthetist and same-day nursing staff. Experienced clinical judgment – that takes into account the underlying comorbidities of a patient, anesthetic technique employed, difficulty of the anticipated thyroidectomy, and risk for postoperative bleeding – is necessary to identify patients suitable for outpatient thyroidectomies. We have established that careful patient selection is essential when deciding whether a patient can be managed as an outpatient. Meticulous hospital structures and organization are integral to successful outpatient thyroidectomies. As noted by Stavrakis and colleagues, increased surgeon experience with thyroidectomy leads to improved postoperative results and lower complication rates (57), this suggests that outpatient thyroidectomy should likely be performed at high volume institutions and by high volume surgeons. Engaging surgical, anesthetic, and administrative stakeholders is pivotal in implementing novel hospital program which facilitates outpatient thyroid surgery as its execution requires sufficient resources and collaboration.

As alluded to by the ATA, appropriate patient selection for ambulatory thyroidectomies requires meticulous implementation of standardized protocol in the preoperative, intraoperative, and postoperative phases of thyroidectomies.

### Preoperative Patient Selection

To ensure appropriate patient selection for ambulatory thyroid surgery, patients should be required to present to a preoperative clinic visit or telehealth visit. Eligibility criteria for outpatient thyroidectomy is outlined in **Table 2** **(** 1). The eligibility criteria are to be reviewed by an anesthesiologist and a registered nurse. Of note, there was variability in the acceptable ASA score cut-off for patient consideration for outpatient thyroidectomy. Previous literature has demonstrated that there is a lack of interrater reliability in the assignment of ASA scores (58). The hypothesized contributing factors behind this is multifactorial, including: anesthetist performance evaluation, resource allocation, and financial reimbursement. As such, we have opted to take a more conservative approach and chose an ASA score of 2 or below in the proposed eligibility criteria.

**Table 2:** Patient selection criteria for outpatient thyroidectomy.

Patient Factors
No major co-morbidities, ASA score of 2 or less
No current pregnancy
BMI <30 and no known diagnosis of obstructive sleep apnea
No neurological (visual or auditory) impairment or psychiatric disorders that could impair communication and/or compliance with instructions
No known medically irreversible bleeding diatheses
Patient referred to the Surgical Pre-admission Clinic and achieved clearance for outpatient surgery
*Social Factors*
Patient willing to participate
Patient has adequate support at home (presence of a caregiver for 48 hours)
Patient and caregiver understanding of pre-operative education
Transportation availability and patient’s proximity to a tertiary care center hospital (<30 minutes)
*Surgical Factors*
Absence of massive or extensive substernal goiter, no locally advanced cancer

ASA, American Society of Anesthesiologists.

Adapted from the ATA guidelines (1).

### Intraoperative Considerations

Intraoperative considerations involve surgical and anesthetic collaboration. From the anesthetic perspective, it is integral that efforts are made to reduce post-operative nausea and vomiting. This is facilitated by reducing the use of perioperative opioids, and the routine administration of non-sedating anti-emetics (59). Furthermore, total intravenous anesthesia is preferred over volatile agents for same-day thyroidectomies as it carries a lower incidence of post-operative nausea and vomiting (60). For analgesia, the surgeon preference is to avoid non-steroidal anti-inflammatory drugs (NSAIDs) to minimize potential bleeding risk (61). Routine administration of acetaminophen as first-line analgesia is encouraged. Interestingly, recent literature has shown that the use of NSAIDs does not increase the risk of cervical bleeding after thyroid and parathyroid surgery. This may have an impact on future post-operative analgesia choices (62). From a surgical perspective, the focus is on visualizing the recurrent laryngeal nerve and utilizing intraoperative neuromonitoring in more complex cases, local analgesia, and hemostasis. It is suggested to utilize intra-operative nerve monitoring or stimulation for recurrent laryngeal nerve integrity confirmation for more complex surgical cases – as is the case with large goiters and completion thyroidectomies (63, 64). Furthermore, intra-operative nerve monitoring can provide real-time feedback with regards to the depth of anesthetic (65); which also has important implications on post-operative nausea and vomiting (66). The routine administration of long-acting local analgesic incision infiltration and cervical block for optimal postoperative pain management is recommended, as is the use of intraoperative hemostatic agents. Cervical blocks have also been shown to be a helpful adjunct to general anesthesia and reduce postoperative use of narcotics for pain (67, 68). This is invaluable in reducing the outpatient opioid prescription burden for these patients.

### Post-Operative Protocol

After surgery, patients should be observed and monitored for 4-8 hours. Same-day surgery nursing staff should be specifically alerted to monitor for potential airway, bleeding, or wound management concerns. Furthermore, parathyroid hormone (PTH) and calcium levels must be drawn for total or completion thyroid surgery in the post-anesthetic care unit to assess for potential hypocalcemia prior to discharge. Discharge eligibility is to be determined by a standardized set of discharge criteria that ensures no evidence of bleeding, swelling, dyspnea, dysphagia, or dysphonia in addition to the usual criteria. Most importantly, clear discharge instructions, and reasons to return to the emergency department, must be provided to the patient and their caregiver to mitigate the risk of later-onset complications. Furthermore, for patients who live further away, an admission may be mitigated through encouraging overnight stay at a nearby hotel (9).

The benefits of outpatient thyroidectomy are well established in the literature; however, maintaining patient safety remains the primary goal when triaging patients for fitness of ambulatory thyroidectomy (33, 34). Thus, before adopting new hospital practices we must assure ourselves that we can maintain patient safety. Our proposed protocol ensures patient safety, satisfaction and provides thorough patient education for post-operative recovery.

## Conclusion

The research surrounding the safety of outpatient thyroidectomies continues to mount with ongoing refinements in selection criteria. Outpatient thyroidectomy, in experienced hands, is safe and is associated with high patient satisfaction and decreased health costs (3, 8). High-volume endocrine surgery centers should consider adopting ambulatory thyroidectomies for the appropriately-selected patient. Suitable patient selection is integral for successful outpatient thyroidectomy. A thorough review of the literature regarding the considerations for ensuring the safety of outpatient thyroidectomies has been provided while incorporating key points from the recent ATA guidelines. Lastly, a proposed protocol integrating important perioperative considerations for ambulatory thyroidectomies has been provided. The authors acknowledge the time commitment and numerous hospital considerations with regards to the implementation of new procedural protocol; however, this is an opportunity to increase safe outpatient thyroidectomies which ultimately better serves our healthcare system and our patients.

## Author Contributions

JP carried out the study, analyzed the dataset, and drafted the manuscript. EB participated in the design of the study and drafted the manuscript. CN, JDP, KH, JF, and AC revised the manuscript and provided expertise feedback. AE participated in the design of the study, contributed to the conception of the study, and revised the manuscript. All authors contributed to the article and approved the submitted version.

## Conflict of Interest

The authors declare that the research was conducted in the absence of any commercial or financial relationships that could be construed as a potential conflict of interest.

## Publisher’s Note

All claims expressed in this article are solely those of the authors and do not necessarily represent those of their affiliated organizations, or those of the publisher, the editors and the reviewers. Any product that may be evaluated in this article, or claim that may be made by its manufacturer, is not guaranteed or endorsed by the publisher.
